# Association of *in vitro* measures of clot formation, platelet function, and fibrinolysis with coronary blood flow and clinical outcomes in acute myocardial infarction

**DOI:** 10.3389/fcvm.2026.1801009

**Published:** 2026-05-07

**Authors:** Anna Kalinskaya, Oleg Dukhin, George Rusakovich, Alexandra Anisimova, Elena Maryukhnich, Daria Vorobyeva, Oxana Ivanova, Polina Gavrilina, Yulia Sukhikh, Anna Gumbina, Sofya Zaitseva, Elena Vasilieva

**Affiliations:** 1 Laboratory of Atherothrombosis, Cardiology Department, Federal State Budgetary Educational Institution of Higher Education Russian University of Medicine of the Ministry of Health of the Russian Federation, Moscow, Russia; 2 I.V. Davydovsky City Clinical Hospital, Moscow Department of Healthcare, Moscow, Russia

**Keywords:** clot formation, coronary blood flow, myocardial infarction, spontaneous reperfusion, thrombosis

## Abstract

**Background:**

The patency of the infarct-related artery (IRA) is known to be associated with clinical outcomes in acute myocardial infarction (AMI). It is known that the presence of impaired coronary blood flow in the acute phase of the disease may worsen the course of the AMI. Despite its great importance, the precise determinants of the IRA blood flow remain incompletely understood.

**Methods:**

A total of 255 AMI patients were included in this study: 169 with impaired (TIMI 0-I) and 86 with preserved (TIMI II-III) coronary blood flow. All patients underwent complete hemostasis profiling, including rotational thromboelastometry, impedance aggregometry and thrombodynamics and were assessed for various in- and out-of-hospital AMI-related complications.

**Results:**

TIMI 0-I patients were characterized by an enhanced *in vitro* prothrombotic profile compared with TIMI II-III patients*.* We identified an association between several hemostatic parameters and impaired coronary blood flow (shorter clotting time (CT); higher platelet aggregation values induced by arachidonic acid, ADP and TRAP-6; higher clot amplitudes (A20 and A25), higher clot density (D) and initial clot growth rate (Vi)). We also found that AUC TRAP-6, CT, and Vi were associated with the rate of in-hospital complications, whereas clot amplitude (A25) was linked to the frequency of out-of-hospital complications

**Conclusion:**

The present study identified several hemostatic parameters associated with both IRA patency and clinical course of AMI. These findings indicate a more pronounced prothrombotic state in patients with reduced coronary blood flow, and further research is required to translate these findings into clinical practice.

## Introduction

Proposed back in the mid-nineties century, the division of acute myocardial infarction (AMI) into ST-segment elevation and non-ST-segment elevation myocardial infarction (STEMI and non-STEMI) remains a cornerstone of clinical practice (1, 2). According to the classical concept, STEMI is usually accompanied by occlusive thrombosis of the infarct-related artery (IRA), whereas in non-STEMI, blood flow is pares of the partially preserved (3). This division dictates differences in the treatment strategy for these patients (4).

At the same time, it is known that approximately 15%–30% of STEMI cases are characterized by preserved blood flow in the IRA (TIMI II or TIMI III), meanwhile 25%–30% of non-STEMI patients present with occluded IRA (5–7). The spontaneous reperfusion (SR) of the IRA is accompanied by ST-segment elevation resolution on electrocardiography (ECG) and/or preserved coronary blood flow in the IRA. The development of SR of the IRA is associated with better clinical outcomes in these patients (8–10).

Although various clinical factors predisposing for blood flow preservation in AMI have been previously described, the exact mechanism of this phenomenon is unknown. The key role in the development of occlusive intracoronary thrombosis is usually assigned to the disrupted functioning of the endogenous fibrinolysis (EF) system, which is unable to lyse the thrombus in a timely manner (11). At the same time, there is evidence of reduced platelet reactivity and plasma hemostasis activity in patients with preserved coronary blood flow (12, 13). We previously demonstrated a lower functional state of the endothelium and a more pronounced inflammatory background in patients with TIMI 0-I blood flow in the IRA in comparison to patients with TIMI II-III blood flow (14).

The aim of the present study was to assess global prothrombotic status in AMI patients and its relationship with coronary blood flow and further course of the disease.

## Methods

### Subjects

This observational cohort study was conducted at the I.V. Davydovsky Moscow City Clinical Hospital between January 2017 and October 2022. The study protocol was developed in accordance with the principles of the Declaration of Helsinki and was approved by the local Ethics Committee. All participants provided informed written consent.

A total of 255 AMI patients were included in our study: 212 presented with STEMI and 43 with non-STEMI. The diagnoses of STEMI or non-STEMI were established in accordance with the Fourth Universal Definition of myocardial infarction (2). All patients underwent standard examination, medical treatment, coronary angiography and IRA revascularization according to current ESC guidelines (4). The patency of the IRA was determined by TIMI grade flow classification (15). Patients were divided into subgroups on the basis of impaired (TIMI 0-I; *n* = 169) or preserved (TIMI II-III; *n* = 86) coronary blood flow.

*Inclusion criteria:* STEMI or non-STEMI patients; onset of symptoms during the 24 h before admission; written informed consent. *Exclusion criteria:* age over 90 or under 18 years; more than 24 h from the onset of symptoms; thrombolytic therapy; signs of cardiogenic shock (CS) on admission; acute and chronic infectious diseases and inflammatory processes on admission; severe anemia or ongoing bleeding; pregnancy; history of oncology disease; ambulatory anticoagulant therapy; inability of IRA determination, decision to refrain from PCI. Full details regarding inclusion and exclusion criteria are provided in Supplementary Table S1.

Upon admission before the initiation of percutaneous coronary intervention (PCI), the parameters of clot formation and endogenous fibrinolysis (via impedance aggregometry, thrombodynamics with concomitant fibrinolysis mode and rotational thromboelastometry) were assessed in all patients.

During the hospital stay, patients were assessed for various in-hospital complications of the disease: death, cardiopulmonary resuscitation (CPR), massive intracoronary thrombosis and infusion of glycoprotein (GP) IIb/IIIa inhibitors, acute heart failure (AHF), stent thrombosis, mechanical ventilation, further development of CS. The combination of these events formed an *in-hospital cumulative endpoint*.

During the long-term of the disease (median observation time—10.8 months), the following events were assessed: death, cardiovascular death, repeated AMI, urgent revascularization, and cardiovascular hospitalization. The combination of these events formed the *out-of-hospital cumulative endpoint*.

### Blood sampling

Peripheral venous blood was drawn via a 21-gauge needle in a 4.5-mL tube containing 0.105 M buffered sodium citrate before the administration of unfractionated heparin and primary PCI. Whole blood samples were used for rotational thromboelastometry and impedance aggregometry. Platelet-free plasma was used for the thrombodynamics. In order to obtain platelet-free plasma whole blood was centrifuged twice: on the first step whole blood samples underwent centrifugation at 1,600 g for 15 min at room temperature and, on the second step, at 10.000 g for 15 min at room temperature.

### Rotational thromboelastometry

The study was conducted using Rotem Delta analyzer according to the manufacturer protocol (The Tem Innovations GmbH, Germany) in the NATEM mode. The following parameters were recorded: clotting time (CT, s), a-angle (*α*,°), clot formation time (CFT, s), thrombus amplitude at different time sections of the study (A10, A20, A25, A30, mm), maximum clot firmness (MCF, mm), and maximum lysis (ML, %).

### Impedance aggregometry

The study was conducted using Multiplate analyzer according to the manufacturer protocol (Werfen, Switzerland). The intensity of platelet aggregation was estimated by measuring the area under the curve (AUC). We assessed platelet aggregation induced by arachidonic acid (AUC ASA, U), adenosine diphosphate (AUC ADP, U) and thrombin receptor activating peptide-6 (AUC TRAP-6, U).

### Thrombodynamics with concomitant fibrinolysis mode

The study was conducted using a T-2 Thrombodynamics analyzer (Hemacore, Russia) according to a standard technique. The following parameters of clot growth were used: clot growth rate (V, µm/min), initial clot growth rate (Vi, µm/min), clot density (D, a.u.), clot size (CS, µm), and spontaneous clot formation time (Tsp, min). A standard activator with urokinase was added to induce thrombus lysis. The lysis was characterized by the lysis index (Li, %), time of lysis onset (LOT, min), clot lysis time (CLT, min), expected lysis time (LTE, min), and rate of lysis progression (LP, %/min).

### Statistical analysis

Statistical analyses were conducted using R version 4.2.1. The data had missing values; therefore, we performed pairwise deletion, excluding the missing values from the analysis. Other values derived from this study underwent normality assessment using the Shapiro–Wilk test, which revealed predominantly non-normal distributions. Comparative analyses involving multiple groups employed the non-parametric Mann–Whitney rank test. Categorical parameters were evaluated via a two-tailed Fisher's exact test using 2 × 2 frequency tables. A significance threshold of *p* ≤ 0.05 was predetermined to determine statistical significance.

To assess the association between baseline parameters and the primary outcome, we conducted a series of univariate logistic regression analyses. Before modeling, all continuous predictor variables were robustly standardized to facilitate the comparison of effect sizes across different scales. This standardization was performed by subtracting the median value of each predictor from its individual values and then dividing by its interquartile range (IQR). The results are presented as Standardized Odds Ratios (SORs), which in this context represent the change in odds for the outcome per one-IQR increase in the predictor variable. All SORs are presented with their corresponding 95% confidence intervals (CIs).

## Results

### Baseline clinical characteristics

In total, 255 patients were included in the current study and stratified by IRA blood flow on initial angiography: TIMI 0-I (*n* = 169) and TIMI II-III (*n* = 86). Age and sex distribution were comparable between groups (Table 1). Dyslipidemia was more frequent in the TIMI 0-I group [69 (42%) vs. 24 (28%); *p* = 0.040] compared to TIMI II-III group. Pre-hospital antiplatelet use did not differ between groups, whereas among loading P2Y12-inhibitor therapy, prasugrel prescription was higher in TIMI 0-I group compared to TIMI II-III group [15 (9%) vs. 1 (1%); *p* = 0.010].

**Table 1:** Baseline clinical characteristics.

Parameter	TIMI 0-I (*n* = 169)	TIMI II-III (*n* = 86)	*p*-value
Clinical data			
Age, years Med [Q1; Q3]	60 [53–68]	62 [54–68]	0.316
Male sex, *n* (%)	126 (75)	68 (79)	0.380
Arterial hypertension, *n* (%)	151 (90)	77 (90)	1.000
Tobacco smoking, *n* (%)	76 (48)	39 (48)	1.000
Diabetes mellitus, *n* (%)	26 (16)	13 (15)	1.000
Dyslipidemia, *n* (%)	69 (42)	24 (28)	0.040
Angina pectoris, *n* (%)	42 (25)	30 (35)	0.140
History of MI, *n* (%)	16 (10)	15 (17)	0.100
History of PCI, *n* (%)	20 (12)	8 (9)	0.670
Atrial fibrillation, *n* (%)	8 (5)	1 (1)	0.280
Stroke, *n* (%)	9 (5)	4 (5)	1.000
Acetylsalicylic acid, *n* (%)	22 (14)	11 (13)	1.000
Beta-blocker, *n* (%)	26 (17)	19 (23)	0.300
Statin, *n* (%)	15 (10)	11 (13)	0.510
Clopidogrel, *n* (%)	0 (0)	2 (3)	0.150
Ticagrelor, *n* (%)	3 (3)	2 (3)	1.000
ACE inhibitor, *n* (%)	26 (18)	19 (23)	0.390
AT-I receptor blocker, *n* (%)	21 (19)	13 (21)	0.690
Calcium channel blocker, *n* (%)	10 (10)	11 (20)	0.140
Physical examination			
Heart rate, beats Med [Q1; Q3]	76 [68; 86]	78 [70; 86]	0.924
Systolic arterial blood pressure, mm Hg Med [Q1; Q3]	140 [130; 160]	144 [135; 160]	0.305
Dyastolic arterial blood pressure, mm Hg Med [Q1; Q3]	84 [70; 90]	80 [75; 95]	0.873
Ongoing angina on admission, *n* (%)	99 (88%)	42 (72%)	0.02
Acute heart failure, *n* (%)	17 (10)	3 (3)	0.080
Pre-hospital therapy			
Acetylsalicylic acid, *n* (%)	148 (91)	75 (91)	1.000
Clopidogrel, *n* (%)	110 (67)	50 (61)	0.320
Ticagrelor, *n* (%)	3 (3)	2 (3)	1.000
Morphine, *n* (%)	49 (30)	17 (21)	0.130
Loading P2Y12 inhibitor before PCI			
Clopidogrel, *n* (%)	13 (8)	13 (15)	0.090
Ticagrelor, *n* (%)	16 (71)	59 (69)	0.670
Prasugrel, *n* (%)	15 (9)	1 (1)	0.010
Periprocedural features			
STEMI, *n* (%)	151 (89)	61 (71)	0.010
ST-segment elevation, mm Med [Q1; Q3]	2.3 [1.0–4.0]	1.0 [1.0–2.0]	<0.001
Pain-to-balloon time, min Med [Q1; Q3]	182.5 [125.0–348.8]	240.0 [165.0–530.0]	0.108
Door-to-balloon time, min Med [Q1; Q3]	34.5 [30.0–40.0]	35.0 [30.3–40.0]	0.565
Multivessel disease, *n* (%)	80 (49)	45 (54)	0.500
Laboratory data			
Hemoglobin, g/L Med [Q1; Q3]	148 [135–156]	146 [135–157]	0.896
White blood cells, 10^9^/L Med [Q1; Q3]	10.2 [8.4–12.3]	9.4 [7.3–11.0]	0.003
Platelets, 10^9^/L Med [Q1; Q3]	241.5 [208.0–291.3]	222.0 [190.0–264.8]	0.010
Neutrophil-to-lymphocyte ratio	3.9 [2.3–5.7]	3.8 [2.7–4.5]	0.836
Creatinine, µmol/L Med [Q1; Q3]	92.0 [79.0–106.0]	89.5 [76.8–102.5]	0.591
Total cholesterol, mmol/L Med [Q1; Q3]	5.7 [4.9–6.7]	5.5 [4.7–6.5]	0.238
High density lipoprotein, mmol/L Med [Q1; Q3]	1.2 [1.0–1.4]	1.3 [1.1–1.5]	0.477
C-reactive protein, mg/L Med [Q1; Q3]	5.0 [2.7–12.9]	3.8 [1.1–7.3]	0.154
Troponin I, ng/L Med [Q1; Q3]	103.0 [42.6–171.0]	23.6 [6.3–75.5]	0.002
Echocardiography			
LV EF, % Med [Q1; Q3]	51 [44–57]	55 [47–60]	0.007
End-diastolic left ventricular volume, mL Med [Q1; Q3]	110 [97–142]	112 [88–131]	0.189
The presence of hypokinesis/akinesis zones of the LV myocardium, *n* (%)	131 (82,8%)	53 (61,6%)	0.041

MI, myocardial infarction; ACE, angiotensin-converting enzyme; AT-I, angiotensin-I; STEMI, ST segment elevation myocardial infarction; PCI, percutaneous coronary intervention; LV EF, left ventricle ejection fraction. Quantitative data are presented as median and interquartile range, and qualitative data are presented as absolute number and percentage of the total number of patients in the group.

TIMI 0-I patients presented more often with STEMI [151 (89%) vs. 61 (71%); *p* < 0.010] and had greater ST-segment elevation on admission [2.3 (1.0–4.0) vs. 1.0 (1.0–2.0) mm; *p* < 0.001]. Ongoing angina on admission was also more common in TIMI 0-I group [99 (88%) vs. 42 (72%); *p* = 0.020].

Laboratory profiles showed higher white blood cell count [10.2 (8.4–12.3) vs. 9.4 (7.3–11.0) *10^9^/L; *p* = 0.003], platelet count [241.5 (208.0–291.3) vs. 222.0 (190.0–264.8) *10^9^/L; *p* = 0.010] and troponin I level [103.0 (42.6–171.0) vs. 23.6 (6.3–75.5) ng/L; *p* = 0.002] in TIMI 0-I group compared to TIMI II-III group. On echocardiography, left ventricular ejection fraction was lower in TIMI 0-I [51 (44–57) vs. 55 (47–60) %; *p* = 0.007].

### Rotational thromboelastometry

**Figure 1:**
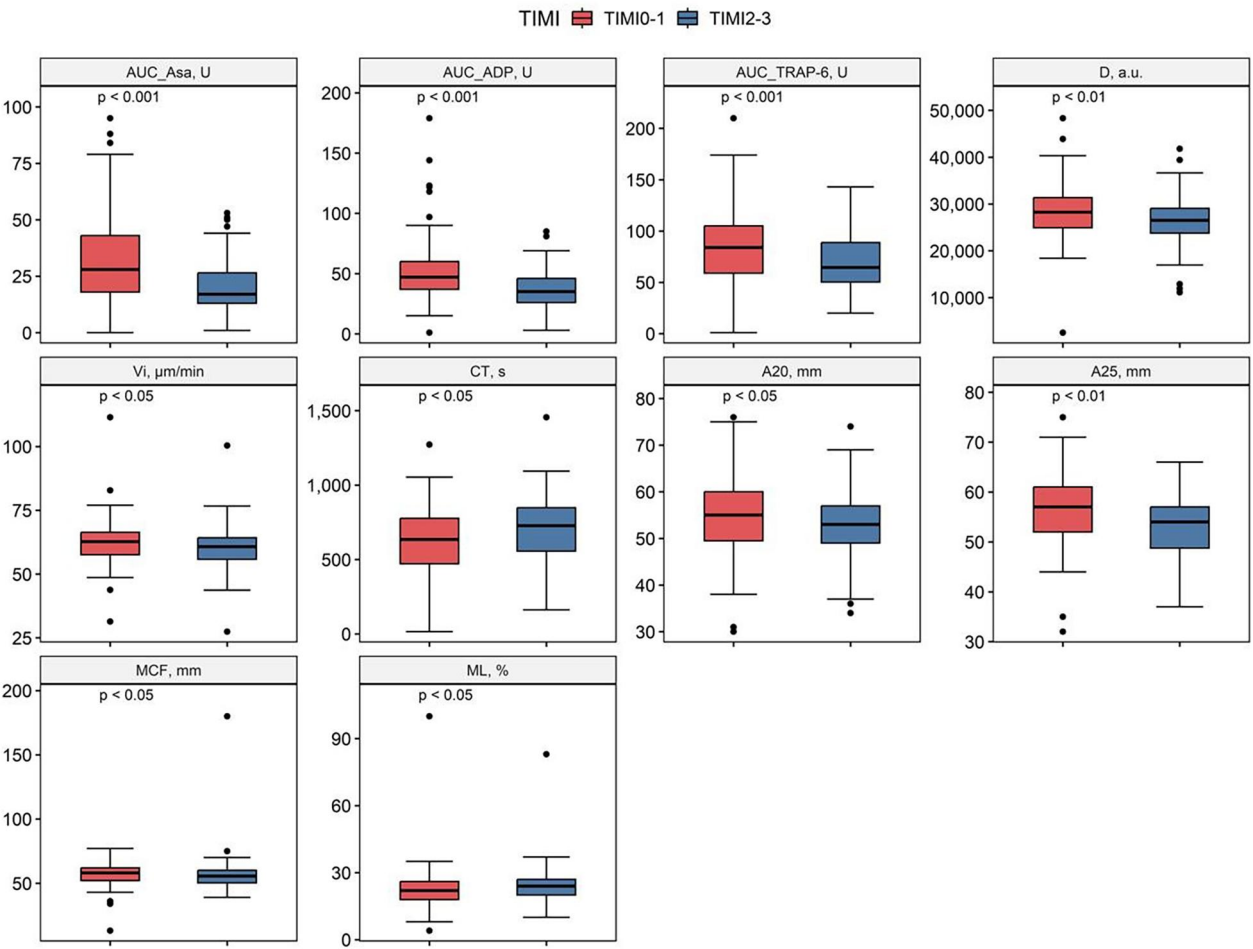
Comparison of selected clot formation parameters in different patient subgroups; AUC ADP, intensity of adenosine diphosphate-induced platelet aggregation; AUC ASA, intensity of arachidonic acid-induced platelet aggregation; AUC TRAP-6, intensity of thrombin receptor activating peptide-6-induced platelet aggregation; D, clot density (thrombodynamics); V, clot growth rate (thrombodynamics); Vi, initial clot growth rate (thrombodynamics); CT, clotting time (rotational thromboelastometry); A20, A25, clot amplitude at different time sections of the study (rotational thromboelastometry); MCF, maximum clot firmness (rotational thromboelastometry), ML, maximum lysis (rotational thromboelastometry).

Patients with TIMI 0-I blood flow demonstrated shorter clotting time [CT, s: 634.0 (471.0–776.5) vs. 727.5 (555.5–847.8); *p* = 0.015], greater clot firmness [MCF, mm: 58.0 (52.0–62.0) vs. 55.5 (50.3–60.0); *p* = 0.020] and amplitude [A20, mm: 55.0 (49.5–60.0) vs. 53.0 (49.0–57.0); *p* = 0.038; A25, mm: 57.0 (52.0–61.0) vs. 54.0 (48.8–57.0); *p* = 0.010] compared with the TIMI II-III group. TIMI 0-I group also had reduced fibrinolytic activity with lower maximum lysis [ML, %: 22.0 (18.0–26.0) vs. 24.0 (20.0–27.0); *p* = 0.034] compared with the TIMI II-III group (Figure 1, Supplementary Table S2).

### Thrombodynamics

TIMI 0-I patients were characterized by increased initial clot growth rate [Vi, µm/min; 62.7 (57.6–66.4) vs. 60.8 (55.8–64.2); *p* = 0.017] and clot density [D, a.u.; 28,259.5 (24,951.8–31,383.5) vs. 26,512.5 (23,800.3–29,083.5); *p* = 0.007] compared with TIMI II-III patients (Figure 1; Supplementary Table S3).

### Impedance aggregometry

TIMI 0-I patients also had increased values of platelet aggregation induced by arachidonic acid [AUC ASA, U; 28.0 (18.0–43.0) vs. 17.0 (13.0–26.5); *p* < 0.001], ADP [AUC ADP, U; 47.0 (37.0–60.0) vs. 35.0 (26.0–46.0); *p* < 0.001] and TRAP-6 [AUC TRAP-6, U; 84.0 (59.0–105.0) vs. 64.5 (50.3–88.8); *p* < 0.001] compared to patients with TIMI II-III blood flow (Figure 1).

### Predictors of coronary blood flow

**Figure 2:**
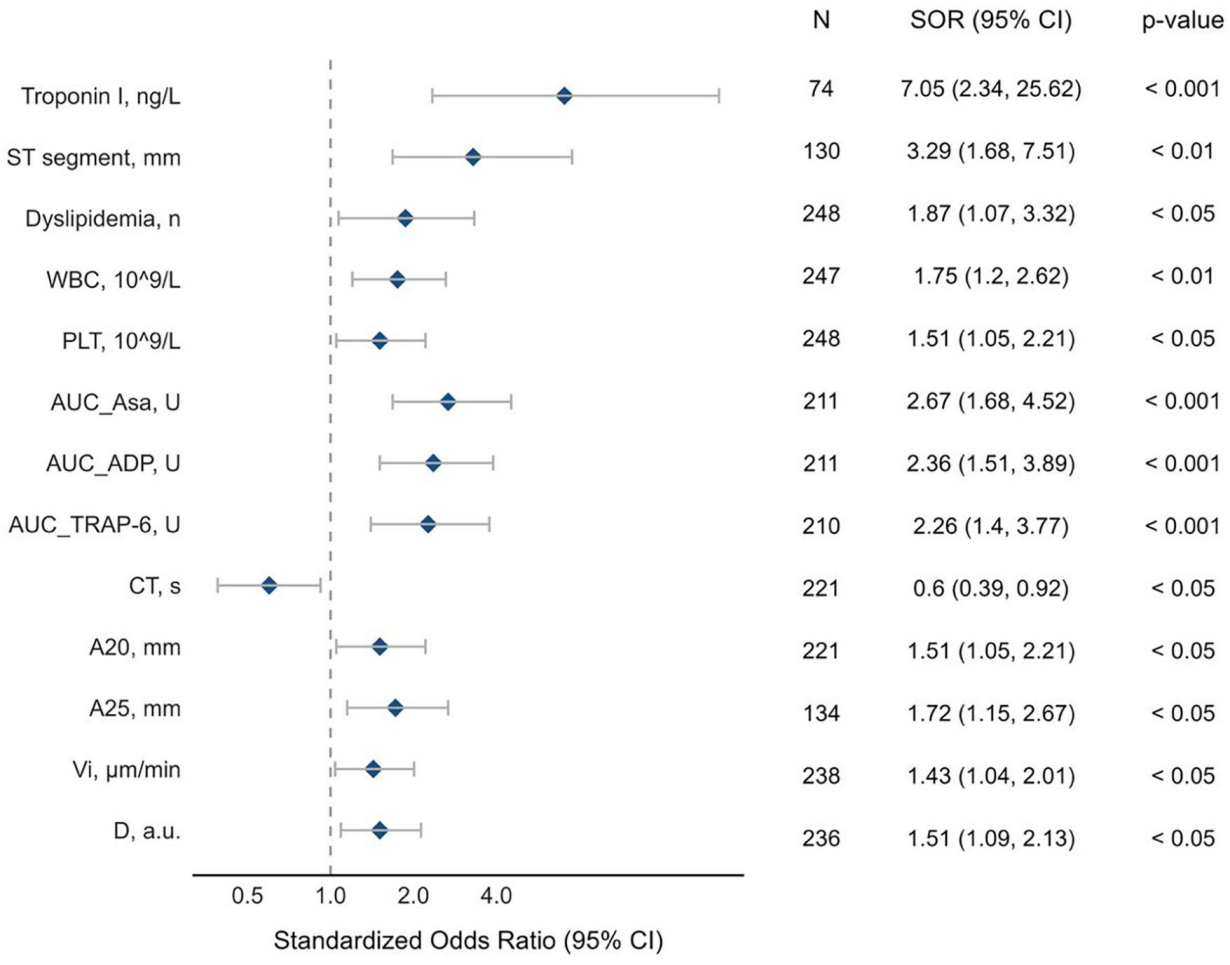
Predictors of coronary blood flow. Within the forest plot, log odds ratios depict the influence of individual predictors on reduced (TIMI 0-I) coronary blood flow. Each line within the plot signifies the effect of a univariable predictor on the standardized log odds, presented with mean estimates denoted by closed circles. Whiskers extending from these circles illustrate the 95% confidence intervals around the standardized log odds, capturing the range of plausible values for each predictor's impact. A vertical reference line is plotted at the null hypothesis. CT, clotting time (rotational thromboelastometry); A15, A20, A25, thrombus amplitude at different time sections of the study (rotational thromboelastometry); AUC ADP, intensity of adenosine diphosphate-induced platelet aggregation (impedance aggregometry); AUC ASA, intensity of arachidonic acid-induced platelet aggregation (impedance aggregometry); AUC TRAP-6, intensity of thrombin receptor activating peptide-6-induced platelet aggregation (impedance aggregometry); D, clot density (thrombodynamics); Vi, initial clot growth rate (thrombodynamics); WBC, white blood cells; PLT, platelets.

In univariable regression analysis, we confirmed that several classical predictors were associated with higher odds of TIMI 0-I blood flow. ST segment elevation showed one of the strongest relation [SOR 3.29, 95% CI (1.68–7.51), *p* < 0.01]. Higher troponin I level [SOR 7.05, 95% CI (2.34–25.62), *p* < 0.001], white blood cell count [SOR 1.75, 95% CI (1.20–2.62), *p* < 0.01] and platelet count [SOR 1.51, 95% CI (1.05–2.21), *p* < 0.05] were also linked to TIMI 0-I blood flow (Figure 2).

In addition, several hemostasis parameters were also associated with poor IRA patency. Higher platelet aggregation activity induced by arachidonic acid [SOR 2.67, 95% CI (1.68–4.52), *p* < 0.001], ADP [SOR 2.36, 95% CI (1.51–2.89), *p* < 0.001] and TRAP-6 [SOR 2.26, 95% CI (1.40–3.77), *p* < 0.001] showed association with TIMI 0-I blood flow. According to rotational thromboelastometry results, higher clot amplitudes were linked to TIMI 0-I blood flow [A20: SOR 1.51, 95% CI (1.05–2.21), *p* < 0.05; A25: SOR 1.71, 95% CI (1.15–2.67), *p* < 0.05]. Thrombodynamics parameters were also associated with impaired blood flow: higher clot density [D: SOR 1.51, 95% CI (1.09-2.13), *p* < 0.05] and initial clot growth rate [Vi: SOR 1.43, 95% CI (1.04–2.01), *p* < 0.05] increased the risk of TIMI 0-I (Figure 2).

### In-hospital complications

Patients with TIMI 0-I blood flow were characterized by a significantly more frequent rate of massive intracoronary thrombosis and GP IIb/IIIa inhibitors infusion and the frequency of registration of an in-hospital cumulative endpoint (Table 2).

**Table 2:** In-hospital complications of AMI.

Parameter	TIMI 0-I (*n* = 169)	TIMI II-III (*n* = 86)	*p*-value
AHF	17 (10)	3 (3)	0.080
Mechanical ventilation	6 (4)	2 (2)	0.720
Massive intracoronary thrombosis and infusion of GP IIb/IIIa inhibitors	32 (19)	7 (8)	0.030
CPR	8 (5)	4 (5)	1.000
Development of CS	6 (4)	3 (3)	1.000
Stent thrombosis	10 (6)	3 (3)	0.550
Death	3 (3)	1 (2)	1.000
In-hospital cumulative endpoint	49 (29)	13 (15)	0.020

AHF, acute heart failure; GP IIb/IIIa, glycoprotein IIb/IIIa; CPR, cardiopulmonary resuscitation; CS, cardiogenic shock; MCS, mechanical circulatory support. Data are presented as absolute number and percentage of the total number of patients in the group, unless otherwise specified. Data are presented as absolute number and percentage of the total number of patients in the group.

**Figure 3:**
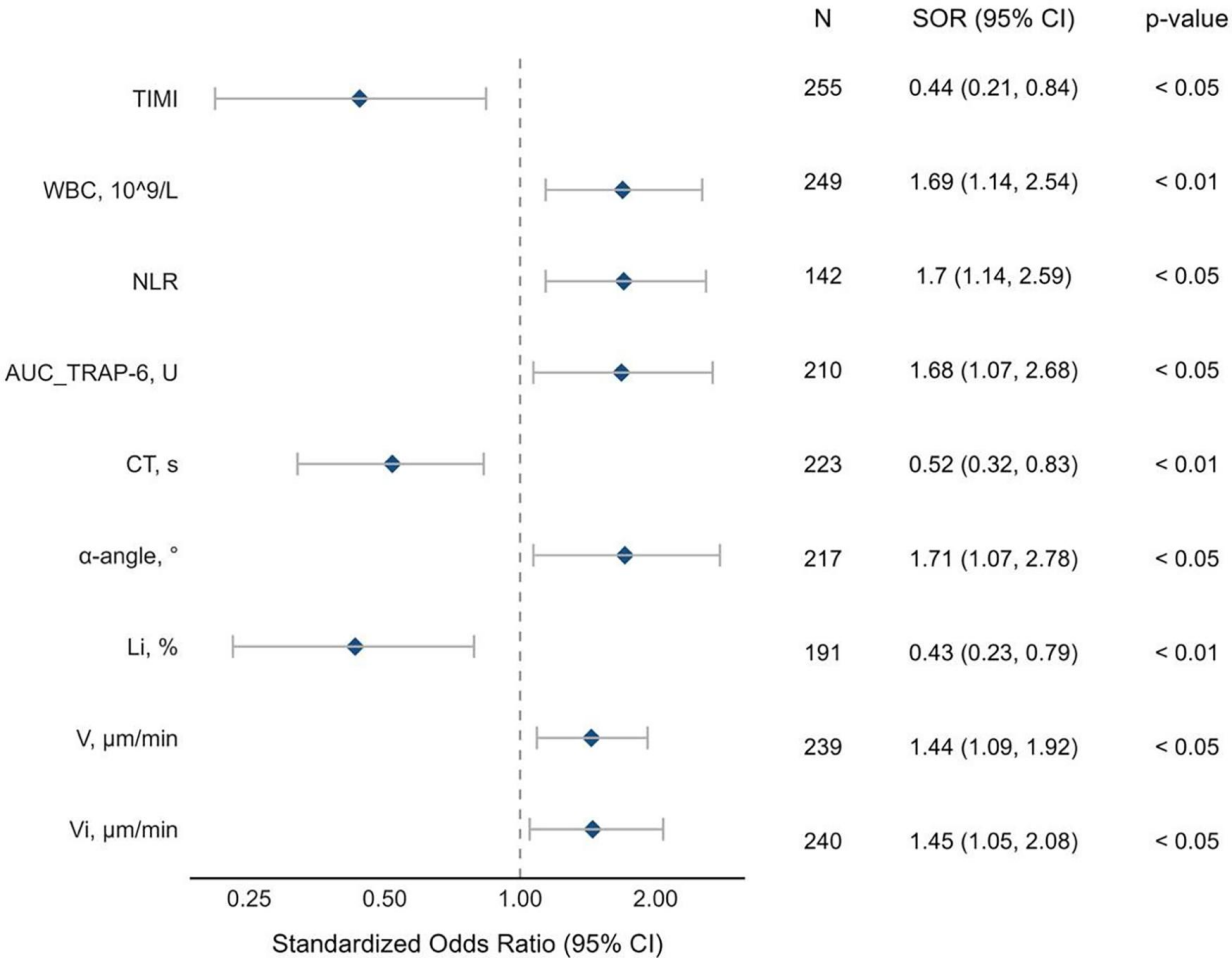
Predictors of the in-hospital complications of AMI. The forest plot displays standardized log odds ratios indicating the impact of individual predictors on in-hospital cumulative ischemic endpoints. Each line represents a predictors effect as standardized long odds, with mean estimates shown as closed circles and whiskers represent 95% confidence intervals. CT, clotting time (rotational thromboelastometry); å, alpha-angle (rotational thromboelastometry); Li, lysis index (Thrombodynamics); AUC TRAP-6, intensity of thrombin receptor activating peptide-6-induced platelet aggregometry (impedance aggregometry); V, clot growth rate (thrombodynamics); Vi, initial clot growth rate (thrombodynamics); NLR, neutrophil-to-lymphocyte ratio; WBC, white blood cells.

Several classic risk factors such as pre-PCI TIMI flow [SOR 2.29, 95% CI (1.19-4.67), *p* < 0.05], age [SOR 1.57, 95% CI (1.05–2.36), *p* = 0.029], white blood cell count [SOR 1.68, 95% CI (1.13–2.52), *p* < 0.01], creatinine level [SOR 1.283, 95% CI (1.036–1.705), *p* = 0.057],NLR [SOR 1.70, 95% CI (1.14–2.59), *p* < 0.05] were associated with in-hospital endpoint. We also found that several parameters of clot formation and lysis increase the risk of in-hospital complications, among which are CT [SOR 0.52, 95% CI (0.32;0.84), *p* < 0.01], a [SOR 1.67, 95% CI (1.04;2.73), *p* < 0.05], Vi [SOR 1.44, 95% CI (1.08;2.07), *p* < 0.05], V [SOR 1.43, 95% CI (1.08–1.92), *p* < 0.05], Li (SOR 0.45, 95% CI [0.24–0.82], *p* < 0.01, AUC TRAP-6 [SOR 1.68, 95% CI (1.07–2.68), *p* < 0.05] (Figure 3). Furthermore, we found out that such parameters as AUC TRAP-6, CT and Vi were common predictors for both TIMI blood flow and in-hospital ischemic endpoints.

### Long-term complications

The final analysis of the long-term period of the disease was available for 160 patients (106 with TIMI 0-I and 54 with TIMI II-III). There was a higher rate of repeated urgent revascularization and a tendency toward a higher rate of out-of-hospital cumulative endpoint among patients with TIMI 0-I blood flow (Table 3).

**Table 3:** Long-term complications of AMI.

Parameter	TIMI 0-I (*n* = 106)	TIMI II-III (*n* = 54)	*p*-value
AMI	2 (2)	2 (3)	0.610
Urgent revascularization	24 (23)	5 (8)	0.030
Cardiovascular hospitalization	28 (25)	8 (14)	0.110
Cardiovascular death	1 (1)	1 (2)	1.000
Death	3 (3)	1 (2)	1.000
Out-of-hospital ischemic endpoint	23 (29)	9 (15)	0.060

AMI, acute myocardial infarction. Data are presented as absolute number and percentage of the total number of patients in the group.

**Figure 4:**
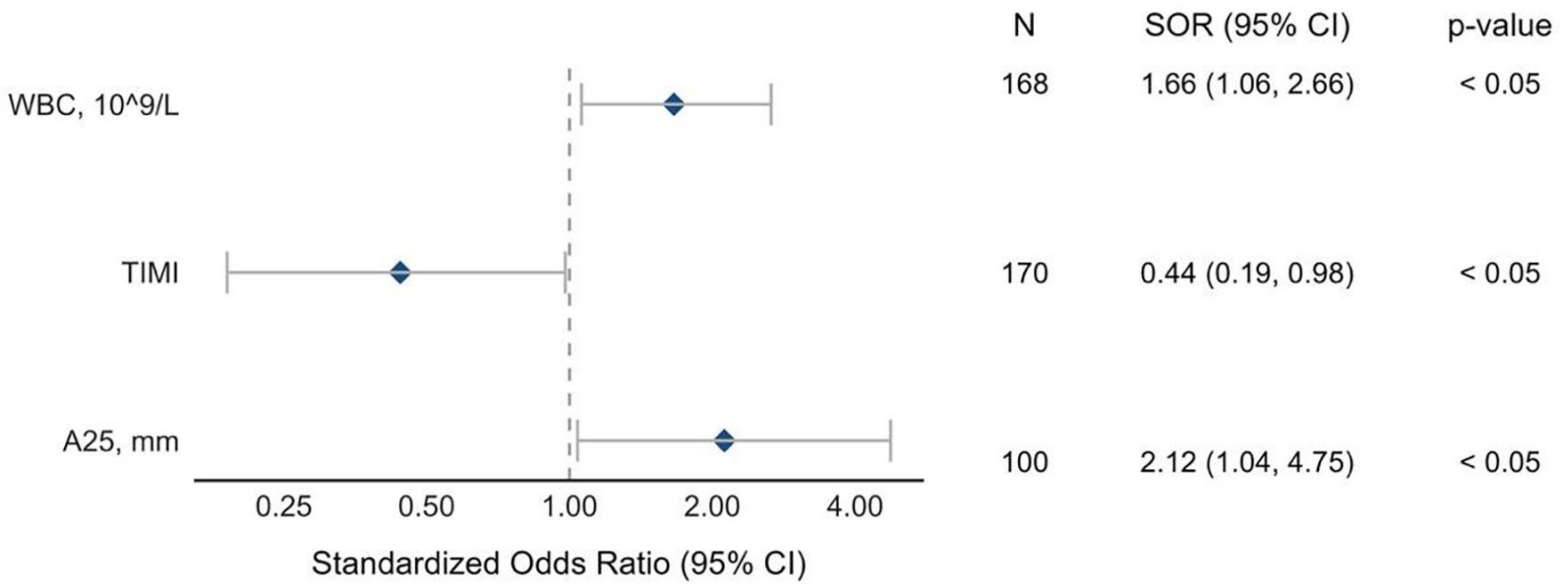
Predictors of the out-of-hospital ischemic complications. The forest plot displays standardized log odds ratios indicating the impact of individual predictors on out-of-hospital ischemic complications. Each line represents a predictor effect as standardized long odds, with mean estimates shown as closed circles and whiskers representing 95% confidence intervals; A25, thrombus amplitude at different time sections of the study (rotational thromboelastometry); WBC, white blood cells.

The risk of cumulative out-of-hospital endpoint was increased in patients with higher WBC count [SOR 1.66, 95% CI (1.06–2.66), *p* < 0.05] and reduced pre-PCI TIMI blood flow [SOR 1.66, 95% CI (1.06–2.66), *p* < 0.05] (Figure 4). Another factor associated with an increased risk of out-of-hospital ischemic events was clot amplitude A25 [SOR 2.12, 95% CI (1.04–4.75, *p* < 0.05)], which, as shown above, was also a TIMI blood flow predictor by itself.

## Discussion

Multiple studies since the late 20th century have shown the association of impaired coronary blood flow in the IRA with worse outcomes for AMI patients (5, 16, 17). Our research reaffirms this finding, demonstrating that reduced coronary blood flow is linked to larger infarct sizes and a negative disease course.

Risk stratification in AMI patients necessitates precise determination of coronary perfusion status. The conventional paradigm in AMI management associates STEMI with initial occlusion of the IRA and partially preserved blood flow with non-STEMI (3). However, this correlation is not absolute, as evidenced by frequent angiographic observations (5, 6, 18). The restoration of blood flow in the IRA is a multifactorial process. A number of predisposing clinical factors have been previously identified, such as younger age, the absence of significant comorbidities (e.g., diabetes mellitus, obesity), lower total cholesterol and lipoprotein (a) levels (8, 19–21). To date, a reliable universal biomarker or parameter for detecting impaired flow in the IRA remains an unmet clinical need. This research was designed to elucidate the factors associated with a reduced blood flow in the IRA in AMI patients.

Our initial findings identified an association between several hemostatic parameters and impaired coronary blood flow (shorter clotting time (CT); higher platelet aggregation values induced by arachidonic acid, ADP and TRAP-6; higher clot amplitudes (A20 and A25) as well as higher clot density (D) and initial clot growth rate (Vi)).

As it was shown previously, the formation of intracoronary thrombosis during AMI is closely associated with the functional state of plasma hemostasis and platelet reactivity (22). A number of research groups have previously demonstrated that patients with a preserved blood flow in the IRA exhibit higher prothrombotic activity and platelet reactivity, alongside reduced activity of endogenous fibrinolysis (12, 13, 23). These observations are further supported by a recent study by Kanji et al. (24), which found an association between higher platelet reactivity, lower activity of endogenous fibrinolysis, and higher frequency of adverse cardiovascular events in patients with TIMI 0-2 blood flow in the IRA (24). Our current results, as well as our previous findings, reconfirmed these data (14, 25).

According to the existing data, TIMI blood flow serves as a prognostic indicator for both short-term (in-hospital) and long-term adverse outcomes (17, 26). In the current study we also demonstrated that reduced blood flow (TIMI 0-I) was associated with in- and out-of-hospital AMI complications. That is why our next step was to establish the association between hemostatic markers of reduced coronary blood flow and the course of the disease. We found that AUC TRAP-6, CT, and Vi were associated with in-hospital complications. In addition, clot amplitude (A25) was linked to out-of-hospital complications. In the context of AMI, modulation of arterial thrombosis and IRA patency through contemporary antithrombotic strategies is a key determinant of clinical outcomes, as emphasized in current ACS guidelines, while experimental data also indicate that pharmacological influences on coagulation and fibrin architecture can modify thrombotic risk and clot stability (4, 27). Our findings suggest that modulation of global hemostatic status may influence not only coronary blood flow in the acute phase of AMI but also the longer-term clinical course, while similar principles have been reported in venous thromboembolism, where attenuation of overall thrombotic burden has been associated with improved survival (28–30).

Thus, our results indicate that certain hemostatic parameters serve as independent predictors of both TIMI blood flow and AMI complications. Based on these findings, it can be hypothesized that the prognostic influence of these hemostatic parameters on the disease complications is mainly related to their role in determining blood flow.

The next finding in our study was the association between white blood cell count and both TIMI flow grade in the IRA and AMI-related complications. Inflammation is a well-established driver of atherothrombosis. It is widely accepted that inflammation in AMI is localized at two sites: the disrupted atherosclerotic plaque and the myocardial necrosis area. Each of these inflammation types can potentiate the development of further AMI complications (31). The study by Börekçi et al. (32) and others (32, 33) demonstrated that both oxidative stress and the neutrophil-to-lymphocyte ratio are independent predictors of IRA occlusion in patients with AMI. In a previous study, we similarly showed elevated levels of several proinflammatory cytokines (MDC, MIP-1b, TNF-a) in TIMI 0-I patients (14). In the present study elevated white blood cells count may be explained by the more prominent inflammatory status and more extensive myocardial damage in TIMI 0-I group.

Our data suggest that prothrombotic markers can potentially be used to identify patients with a high risk of AMI-related complications. This information may aid in clinical decision-making in the acute phase of AMI and determining the appropriate duration and intensity of antithrombotic therapy for AMI patients.

### Study limitations

This study has several limitations. First, it was conducted in a single, high-volume tertiary center, which may limit the generalizability of our findings to other settings and healthcare systems. Second, the observational cohort design precludes any causal inferences and leaves room for residual confounding. Third, the sample size, although moderate, may still be insufficient to detect more subtle associations, particularly in subgroup analyses and for less frequent clinical endpoints. Finally, while several hemostatic parameters were associated with clinical outcomes in univariate analysis, the relatively small number of cumulative ischemic events—particularly in the long-term follow-up—precluded the use of a robust multivariable logistic regression model. Future studies with larger cohorts are required to determine the independent prognostic value of these markers and to identify which specific parameters provide the highest predictive accuracy.

## Conclusion

The present study identified several hemostatic parameters associated with both the IRA patency and the clinical course of AMI. The association with both reduced TIMI blood flow and short- and long-term adverse events was demonstrated for shorter clotting time (CT) and elevated clot amplitude (A25) on rotational thromboelastometry, higher values of platelets aggregation, induced by TRAP-6, and higher initial clot growth rate (Vi) assessed by thrombodynamics. These findings indicate a more pronounced prothrombotic state in patients with reduced coronary blood flow, which represent one of the most meaningful mechanisms determining the higher complications rate in this group of patients. Further research is needed to investigate the place of these prothrombotic markers in the current clinical practice in AMI patients.

## Data Availability

The datasets presented in this article are not readily available because The study is part of an ongoing research program, and the dataset is currently subject to institutional regulations and ethics committee restrictions. For this reason, the full dataset cannot be shared at this time. Requests to access the datasets should be directed to Oleg Dukhin, Dukhin.o@yandex.ru.
