# Supplementary material for: Association of in vitro measures of clot formation, platelet function, and fibrinolysis with coronary blood flow and clinical outcomes in acute myocardial infarction
Source: Front Cardiovasc Med. 2026 May 7;13:1801009. doi: 10.3389/fcvm.2026.1801009 (PMC13190566; doi:10.3389/fcvm.2026.1801009)
Supplement: Supplementary file 1 [file Datasheet1.docx]

**Supplementary material**

**Table S1** Inclusion and exclusion criteria

| **Inclusion criteria** |
| --- |
| 1. Myocardial infarction diagnosis in accordance with Forth Universal Definition of Myocardial Infarction (2018):   detection of a rise and/or fall of cTn values with at least one value above the 99th percentile URL and at least one of the following:  • Symptoms of myocardial ischaemia;  • New ischaemic ECG changes;  • Development of pathological Q waves;  • Imaging evidence of new loss of viable myocardium or new regional wall motion abnormality in a pattern consistent with an ischaemic aetiology;  • Identification of a coronary thrombus by angiography or autopsy |
| 1. Written informed consent |
| **Exclusion criteria** |
| 1. Age over 90 or under 18 years |
| 1. More than 24 hours from the onset of symptoms |
| 1. Thrombolytic therapy |
| 1. Signs of cardiogenic shock (CS) on admission |
| 1. Acute and chronic infectious diseases |
| 1. Inflammatory processes on admission |
| 1. Severe anemia or ongoing bleeding |
| 1. Pregnancy |
| 1. History of oncology disease |
| 1. Ambulatory anticoagulant therapy |
| 1. Inability of IRA determination |
| 1. Decision to refrain from PCI |

**Table S2** Comparative analyses results: rotational thromboelastometry

| Parameter | TIMI 0-I | TIMI II-III | p |
| --- | --- | --- | --- |
| CT, s | 634.0 [471.0­776.5] | 727.5 [555.5­847.8] | p=0.015 |
| α,° | 53.0 [46.0­62.0] | 52.0 [45.0­58.0] | p=0.407 |
| CFT, s | 229.0 [154.5­301.0] | 217.0 [155.0­285.8] | p=0.663 |
| A10, mm | 43.0 [38.0­51.5] | 42.5 [36.0­48.0] | p=0.236 |
| A20, mm | 55.0 [49.5­60.0] | 53.0 [49.0­57.0] | p=0.038 |
| A25, mm | 57.0 [52.0­61.0] | 54.0 [48.8­57.0] | p=0.01 |
| A30, mm | 58.0 [53.0­62.0] | 55.0 [50.5­60.0] | p=0.111 |
| MCF, mm | 58.0 [52.0­62.0] | 55.5 [50.3­60.0] | p=0.023 |
| ML, % | 22.0 [ 18.0­26.0] | 24.0 [20.0­27.0] | p=0.034 |

**Table S3** Comparative analyses results: thrombodynamics

| Parameter | TIMI 0-I | TIMI II-III | p |
| --- | --- | --- | --- |
| V, µm/min | 36.0 [31.3­43.8] | 35.1 [29.7­39.8] | p=0.171 |
| Vi, µm/min | 62.7 [57.6­66.4] | 60.8 [55.8­64.2] | p=0.017 |
| D, arb units | 28259.5 [24951.8­31383.5] | 26512.5 [23800.3­29083.5] | p=0.007 |
| CS, µm | 1324.0 [1228.0­1459.5] | 1347,0 [1195,0­1410.5] | p=0.477 |
| Tsp, min | 29.5 [20.9­53.9] | 38.3 [23.6­56.7] | p=0.306 |
| Li, % | 51.4 [33.0­56.1] | 51.2 [42.3­56.4] | p=0.936 |
| LOT, min | 30.4 [23.5­40.7] | 31.6 [24.7­43.4] | p=0.360 |
| CLT, min | 25.2 [17.5­34.2] | 28.0 [19.6­34.5] | p=0.266 |
| LTE, min | 26.5 [18.9­37.8] | 30.7 [20.9­39.5] | p=0.400 |
| LP, %/min | 3.8 [2.7­7.1] | 3.9 [2.7­5.7] | p=0.976 |
